# Molecular Signature Expands the Landscape of Driver Negative Thyroid Cancers

**DOI:** 10.3390/cancers13205184

**Published:** 2021-10-15

**Authors:** Larissa Valdemarin Bim, Thaise Nayane Ribeiro Carneiro, Vanessa Candiotti Buzatto, Gabriel Avelar Colozza-Gama, Fernanda C. Koyama, Debora Mota Dias Thomaz, Ana Carolina de Jesus Paniza, Eunjung Alice Lee, Pedro Alexandre Favoretto Galante, Janete Maria Cerutti

**Affiliations:** 1 Genetic Bases of Thyroid Tumors Laboratory, Division of Genetics, Department of Morphology and Genetics, Escola Paulista de Medicina, Universidade Federal de São Paulo, Pedro de Toledo 669, 11 Andar, São Paulo 04039-032, SP, Brazil; larissa.bim@unifesp.br (L.V.B.); thaise.nayane@unifesp.br (T.N.R.C.); gabriel.gama@unifesp.br (G.A.C.-G.); thomaz@unifesp.br (D.M.D.T.); ana.paniza.ext@dasa.com.br (A.C.d.J.P.); 2 Centro de Oncologia Molecular, Hospital Sírio-Libanês, Rua Professor Daher Cutait 69, Bela Vista, São Paulo 01308-060, SP, Brazil; vbuzatto@mochsl.org.br (V.C.B.); fernanda.koyama@ocpmedicine.com (F.C.K.); pgalante@mochsl.org.br (P.A.F.G.); 3 Division of Genetics and Genomics, Boston Children’s Hospital and Harvard Medical School, 3 Blackfan Circle, Boston, MA 02115, USA; ealice.lee@childrens.harvard.edu

**Keywords:** thyroid carcinoma, FTC, FVPTC, HCC, NRAS, BRAF, *NCOR1*, *ECD*, *NUP98*, *SPOP*, expression profile

## Abstract

**Simple Summary:**

Thyroid cancer is the most common endocrine malignancy. However, the cytological diagnosis of certain malignant thyroid tumors and their benign counterparts is a challenge for preoperative diagnosis, as nearly 20–30% of biopsied thyroid nodules are classified as having an indeterminate risk of malignancy. Although multigene panels customized for thyroid cancer include most of the known tumor driver genes, many thyroid samples have negative results for the tested genes. To expand our knowledge, driver-negative samples were profiled by RNA-sequencing. Here, we report novel gene variants that might be associated with the pathogenesis of thyroid tumors, and show that driver-negative samples have two distinct expression signatures. We believe that our findings will ultimately impact preoperative diagnosis on thyroid nodules and provide directions for further experimental validation analysis.

**Abstract:**

Thyroid cancer is the most common endocrine malignancy. However, the cytological diagnosis of follicular thyroid carcinoma (FTC), Hürthle cell carcinoma (HCC), and follicular variant of papillary thyroid carcinoma (FVPTC) and their benign counterparts is a challenge for preoperative diagnosis. Nearly 20–30% of biopsied thyroid nodules are classified as having indeterminate risk of malignancy and incur costs to the health care system. Based on that, 120 patients were screened for the main driver mutations previously described in thyroid cancer. Subsequently, 14 mutation-negative cases that are the main source of diagnostic errors (FTC, HCC, or FVPTC) underwent RNA-Sequencing analysis. Somatic variants in candidate driver genes (*ECD, NUP98,* *LRP1B, NCOR1, ATM, SOS1*, and *SPOP*) and fusions were described. *NCOR1* and *SPOP* variants underwent validation. Moreover, expression profiling of driver-negative samples was compared to 16 BRAF V600E, *RAS*, or *PAX8-PPARg* positive samples. Negative samples were separated in two clusters, following the expression pattern of the *RAS/PAX8-PPARg* or BRAF V600E positive samples. Both negative groups showed distinct BRS, ERK, and TDS scores, tumor mutation burden, signaling pathways and immune cell profile. Altogether, here we report novel gene variants and describe cancer-related pathways that might impact preoperative diagnosis and provide insights into thyroid tumor biology.

## 1. Introduction

Thyroid cancer is the most common endocrine malignancy. Currently, it is the 5th most common cancer in women in the United States and in Brazil [1,2,3]. Differentiated thyroid carcinomas (DTCs) are the most common type of thyroid carcinoma (90%), which can be subdivided into papillary thyroid carcinoma (PTC), follicular thyroid carcinoma (FTC), and Hürthle cell carcinoma (HCC) [4,5].

PTC is the most common thyroid cancer subtype and constitutes more than 80% of DTC. Over ten histologic variants of PTC have been described, classical (CVPTC) and follicular variants (FVPTC) being the most common. Notably, FVPTC is the variant with the highest rate of increase in the last decades [6,7]. The genetic events driving development and progression of PTCs have been extensively studied and the majority of these changes result in constitutive activation of the MAPK signaling pathway. Although the prevalence of *BRAF V600E* mutation varies across populations, in most studies, it is reported as the most prevalent genetic alteration in PTCs. *RET/PTC* fusions are the second most common.

FTC and HCC are two distinct tumor entities that have a more aggressive clinical behavior compared to PTC, but less information regarding molecular events underlying development and progression [6]. The preoperative differential diagnosis between the malignant FTC and HCC and their benign counterparts (follicular thyroid adenoma (FTA) and Hürthle cells adenoma (HCA)) is problematic, with nearly 20–30% of biopsied thyroid nodules still classified as having an indeterminate risk of malignancy and undergoing diagnostic surgery [7,8,9,10].

An overlap of benign and malign lesions also occurs at the molecular level, as mutations in *RAS* and *PAX8-PPARg* genes are identified in both lesions [11,12,13,14]. Additionally, the absence of mutations widely known as drivers of PTC, FTC, and HCC do not exclude malignancy. Therefore, even after incorporating molecular tests into the algorithm for differential diagnosis of thyroid nodules classified as indeterminate or suspicious, it still could generate false positive or false negative results.

To expand the knowledge of the molecular characterization of thyroid carcinomas, which would ultimately improve the sensitivity of molecular tests, The Cancer Genome Atlas (TCGA) consortium analyzed over 400 cases of PTC [15]. However, over 70% of the cases were CVPTC and nearly 27% lacked alterations in the established and recurrent thyroid driver genes. After attributing different genomic regions with copy number alterations as possible drivers for PTC, they still remained with 3.5% of driver negative cases. Additionally, Yoo et al.’s [10] mutational landscape and expression profiling focusing on follicular variants of thyroid tumors showed that FVPTC harbored an intermediate mutational status between CVPTC and minimally invasive FTC; however, it was not able to distinguish between FTA and FTC. Moreover, they found a higher percentage of FTA, FTC, and FVPTC without a known or novel driver gene [10]. Recently, whole genome sequencing analysis was performed in 2 HCC and 11 widely invasive FTC. The authors identified one or several mutations in coding regions of well-established genes rated as top 20 mutated in FTC, while no coding mutations were found in HCC [12].

To discover new candidate driver genes or novel cancer-related pathway leading to thyroid cancer, we performed an RNA-Sequencing (RNA-Seq) analysis in samples that are a source of pre-surgical diagnostic error (FVPTC, FTC, and HCC) and were negative for mutations in genes ranked as most commonly mutated in thyroid cancer. We expect that our findings may help to narrow this knowledge gap and provide directions for further clinical and experimental validation analysis.

## 2. Materials and Methods

### 2.1. Patient Cohort

Thyroid tumor samples were collected from patients who underwent total thyroidectomy at Hospital São Paulo (Universidade Federal de São Paulo—UNIFESP) and Hospital das Clínicas (Universidade de São Paulo—USP), São Paulo, Brazil and snap-frozen in liquid nitrogen within 60 min.

**Figure 1:**
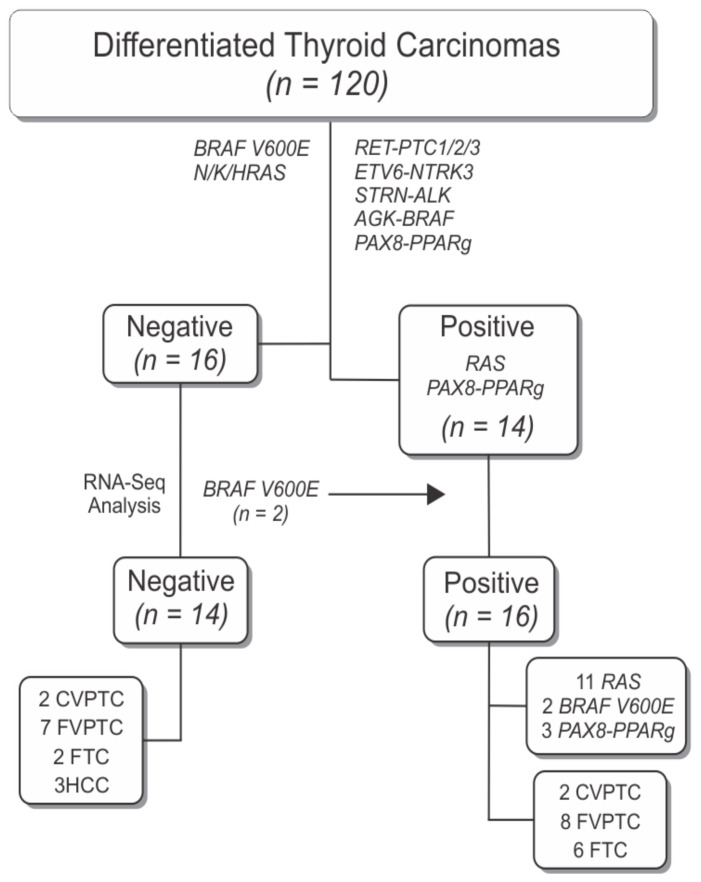
Flowchart with overview of sample selection criteria resulting in the final discovery cohort of 30 samples used for RNA-sequencing analysis. Fourteen samples were positive for *RAS* and *PAX8-PPARg* mutations, which are commonly found in thyroid tumors that are a source of diagnostic error on FNA. Two samples that were considered negative in the initial screening were positive for BRAF V600E by RNA-sequencing analysis and were relocated to the positive group. Fourteen samples were negative for the ranked gene mutations in thyroid carcinomas.

The initial cohort encompassed 120 fresh frozen thyroid tissues from patients diagnosed with DTC. The thyroid tumor samples were screened for somatic mutations in the main driver genes associated with the pathogenesis of DTC (*BRAF* V600E, *H/K/NRAS, RET-PTC1, RET-PTC2, RET-PTC3, ETV6-NTRK3, STRN-ALK, AGK-BRAF*, and *PAX8-PPARg*) [16,17]. Sixteen cases with negative results for the mutations tested were selected to comprise the discovery cohort. For comparative purposes, we included 11 samples positive for *RAS* mutations and 3 samples positive for *PAX8-PPARg* fusion (Figure 1). Clinical-pathological data of the 30 selected patients can be assessed at Table 1.

For validation purposes, an independent cohort, encompassing 53 thyroid tumors (12 PTC, 8 FVPTC, 7 FTC, 7 FTA, 16 HCC, and 3 HCA), was included in the study.

The study was conducted under the approval of the Ethical Committee of the Universidade Federal de São Paulo—UNIFESP/EPM. Informed consent was obtained from each patient after full explanation of the purpose and nature of all procedures used.

### 2.2. RNA Isolation and Library Preparation

RNA isolation and library preparation were conducted as previously described [18] using TrueSeq Stranded mRNA sample preparation kit v.2 (Illumina Inc., San Diego, CA, USA) on an Illumina NextSeq 500 sequencing platform (Illumina Inc.) at the Centro de Oncologia Molecular, Hospital Sírio-libanês, São Paulo, Brazil (Table S1).

### 2.3. Variant Calling and Gene Expression Analysis

The FASTQ file was mapped against the human reference genome for variant calling (GRCh37) or gene expression analysis (GRCh38) using STAR 2-pass mode version 2.7 and transcript abundance was estimated using HTSeq count.

A pipeline was developed for detection of single nucleotide variants (SNVs) and small insertions/deletions (InDels) from RNA-sequencing data. SNVs and InDels were called using the Genomic Analysis Toolkit (GATK) HaplotyperCaller version 3.7.0, as previously described [18].

Variants with poor mapping quality (QC < 20) and Minor Allele Frequency (MAF) > 0.001 in genome aggregation database (gnomAD) or in the Brazilian genomic variants (ABraOM) database [19] were filtered out. Normal thyroid tissue data obtained from the ENCODE dataset (https://www.encodeproject.org, accessed on 20 February 2019) were processed under the same pipeline described above and correspondent annotations were excluded from the tumor mutation results. All filtered out variants in oncogenes or tumor suppressor genes (TSG) reported in COSMIC [20] were reimported. Maftools software [21] was used to analyze and visualize SNVs and Indels. Data from TCGA-THCA was analyzed on cBioPortal software [22,23]. In silico prediction of structural and functional impacts of variants on final protein and protein–protein interactions was accessed through HOPE software [24] and Interactome INSIDER [25]. Damaging impact scores were accessed through SIFT and PolyPhen-2 scores.

### 2.4. Fusion Gene Analysis

Fusion annotation was performed using STAR-Fusion (parameters: –annotation–coding-effect) version 1.3.1 [26]. Filter parameters included fusion fragments per million total reads (FFPM) > 0.1 and Junction Reads Counts and Spanning Fragments Counts > 10. Comparison between groups was made using Maftools software. TCGA fusion data was assessed at Tumor Fusions web portal (http://www.tumorfusions.org, accessed on 12 March 2019) [27]. Fusion transcripts detected in normal thyroid dataset from the ENCODE were filtered out (see variant calling section).

### 2.5. Confirmation of Selected Variants by Sanger Sequencing

The candidate variants were confirmed in the matching RNA used for library preparation by Sanger sequencing. Primers were designed using Primer3 V.0.4.0. cDNA synthesis and PCR reactions were performed as previously described [28]. PCR products were purified and sequenced in an ABI 3130 Genetic Analyser using BigDye terminator kit, as described [28]. Following a validation step in the discovery cohort, the variants were assessed in an independent set of benign and malignant thyroid samples. For this analysis, the DNA was isolated, amplified by PCR, and sequenced as previously described [28]. Primers and PCR conditions are available on request.

### 2.6. Sample Cluster Analysis

Annotated genes were filtered for read count > 10 in at least 90% of samples and genes under any pseudogene-associated class in human reference genome (GRCh37) were excluded to reduce mismapping. To ensure all cancer-related genes expressed in our samples were represented, oncogenes and TSG from COSMIC were reimported. Reads were FPM (fragments per million) normalized through DESeq2 R package (version 1.30.0) [29] and log2 + 1 transformed. A hierarchical clustering showed that one sample (sample 4, Table 1) had a very discrepant pattern of expression compared to the others and was excluded from this analysis. FactoMineR R package (version 2.4) [30] resulted a value of 1-H < 0.5, which indicates that our samples could be clustered. The test was bootstrapped 512 times and the mean values were used.

R package NbClust (version 3.0) [31] was used to verify the optimal number of clusters for many different algorithms. This test was bootstrapped 1080 times with a random selection of 10% of all genes, for 19 different algorithms. This approach defined the optimal number of clusters as two. Next, R package clValid [32] defined the best clustering method as hierarchical. Finally, using the McQuitty method on FactoExtra R package (version 1.0.3) [33], 29 samples were clustered, based on expression pattern, in two clusters with the hierarchical method and visualized in PCA dimensions.

### 2.7. BRAF V600E-RAS Score (BRS)

For score calculation, we used 87 PTC samples from the TCGA-THCA cohort (26 BRAF V600E and 61 RAS), which were downloaded from the TCGA data portal (https://portal.gdc.cancer.gov, accessed on 10 July 2020) and processed according to the pipeline described above. The BRS was calculated as previously described [15]. Briefly, the centroids for BRAF V600E positive samples and RAS positive samples were calculated with K-mean algorithm on the FPM normalized expression of the 71-gene expression signature. The mean expression value for the 71 genes was used to calculate the Euclidean distance of each of the 29 samples from the BRAF and RAS centroids. This value was then scaled from −1 to 1.

### 2.8. Thyroid Differentiation Score (TDS) and ERK Score

The Thyroid Differentiation Score (TDS) and ERK score were calculated using the same parameters as previously determined [15]. The TDS was based on a list of 16 genes related to thyroid function [15]. The ERK score was based on a list of 52 genes from the ERK-related MAPK pathway [34]. Briefly, normalized FPM expression values for selected genes were centered by their median and log2 transformed. The final score was obtained extracting the mean of the selected genes for each sample.

### 2.9. TMB Calculation

The Tumor Mutation Burden (TMB) was calculated using the R package Maftools with a capture size of 50. The TMB for each tumor was obtained by dividing the total number of expressed somatic mutations (non-synonymous variants and InDels) per mega base (Mb) of the region sequenced. The results were pooled into analysis groups.

### 2.10. Differential Gene Expression Analysis

R package DESeq2 (version 1.30.0) was used to detect differentially expressed genes (DEGs) between selected groups, using adjusted *p*-value < 0.05. Negative samples from the discovery cohort were separated in two groups according to results obtained from sample cluster formation (see Sample cluster analysis) and compared against each other. Moreover, as a second comparison group, DEGs between RAS positive samples and negative samples located in the same cluster were assessed.

### 2.11. Differential Expression Analysis on PTC from the TCGA Cohort

The RNA-seq data from 353 PTC samples of the TCGA-THCA cohort (60 RAS and 293 BRAF V600E positive) were downloaded from the TCGA data portal (https://portal.gdc.cancer.gov, accessed on 10 July 2020). DESeq2 was applied for comparison between BRAF V600E and RAS positive groups using *p* adjusted <0.05.

### 2.12. Pathway Enrichment Analysis

Enrichment analysis was performed on DEGs from each comparison group of the discovery cohort and in PTC from the TCGA cohort. Pathway-based analysis was performed using ReactomePA and R/Bioconductor package, which provides a gene set enrichment analysis (GSEA) [35,36]. The R program Gene Set Enrichment Analysis of KEGG (gseKEGG) from R/Bioconductor ClusterProfiler package, which uses information from KEGG pathway database, was also applied [37]. Further, gageData package was used to evaluate specific pathways, shading molecules on KEGG pathway maps according to their degree of dysregulation [38].

### 2.13. Evaluation of Tumor-Infiltrating Immune Cells

To evaluate the fraction of tumor-infiltrating immune cells (TIICs) in the discovery cohort and 353 PTC from TCGA-THCA, we used CIBERSORTx software (https://cibersortx.stanford.edu, accessed on 18 September 2020) [39]. Samples were analyzed at a threshold of *p* < 0.05, 1000 permutations, and LM22 signature provided by the software, with B-mode batch correction and calculation of the absolute score (which reflects the absolute proportion of each cell type).

### 2.14. Statistics

Statistical analysis was made using GraphPad Prism 5 software. Student’s *t*-test was used when pairs of data were analyzed and Chi square test was used for contingency data. All data passed the Shapiro–Wilk normality test.

## 3. Results

### 3.1. RNA-Sequencing Framework

To identify novel genes that might provide additional diagnosis information in patients with nodules classified as indeterminate, we selected histological subtypes that are still a challenge in FNA biopsy and that were negative for main driver mutations found in thyroid carcinomas to undergo RNA-Seq analysis (Figure 1).

We obtained on average 126 million reads per sample (56–242 million reads) with an average of 91.42% (89.2–93.8%) of them correctly mapping to the human reference genome (after Q20 mapping quality score filtering) (Table S1). Variant calling revealed that 2 cases (samples 11 and 12, Table 1), previously classified as negative by Sanger sequencing, were positive for BRAF V600E mutation. Therefore, the final discovery cohort was composed of 14 cases without the key thyroid driver mutations related to DTCs (henceforth called “negative samples”) and 16 cases positive for mutations on common driver genes found in FTC and FVPTC (*H/K/NRAS* and *PAX8-PPARg*) (Figure 1).

### 3.2. Identification of Novel Mutations in Thyroid Cancer

**Figure 2:**
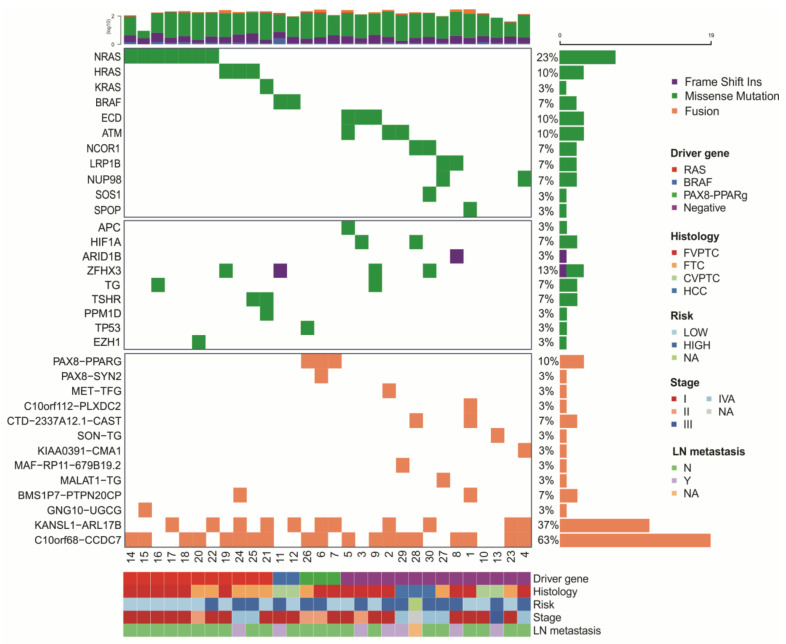
Mutational outline of somatic mutation identified in RNA-sequenced cohort. Each row represents a mutated gene and each column represents one case, as described in Table 1. Upper panel depicts known driver thyroid genes found mutated in 16 positive samples and candidate genes found mutated exclusively on the 14 negative samples; middle panel displays co-occurring mutations in candidate driver genes previously described across PTC, FVPTC, FTC, and FTA samples [10,15]; bottom panel displays main fusions found in the discovery cohort. Top bar plot represents log-transformed sample read count. The right bars indicate variant frequency. Samples are sorted by histological subtype, risk for recurrence, tumor stage, and lymph node (LN) metastasis.

Variant calling following a strict set of filters resulted in 2584 SNVs and InDels in 1805 genes for the 14 negative samples. The mutational landscape of the 30 thyroid carcinomas is illustrated in Figure 2.

Focusing on the 14 samples that were negative for the most frequently thyroid mutated genes, we identified two candidate driver genes (*ECD* and *NUP98*) that were not previously implicated in thyroid cancer and recovered other potential drivers previously described in thyroid tumors (*LRP1B, NCOR1, ATM, SOS1*, and *SPOP*) [14,15,38] (Figure 2—upper panel).

**Figure 3:**
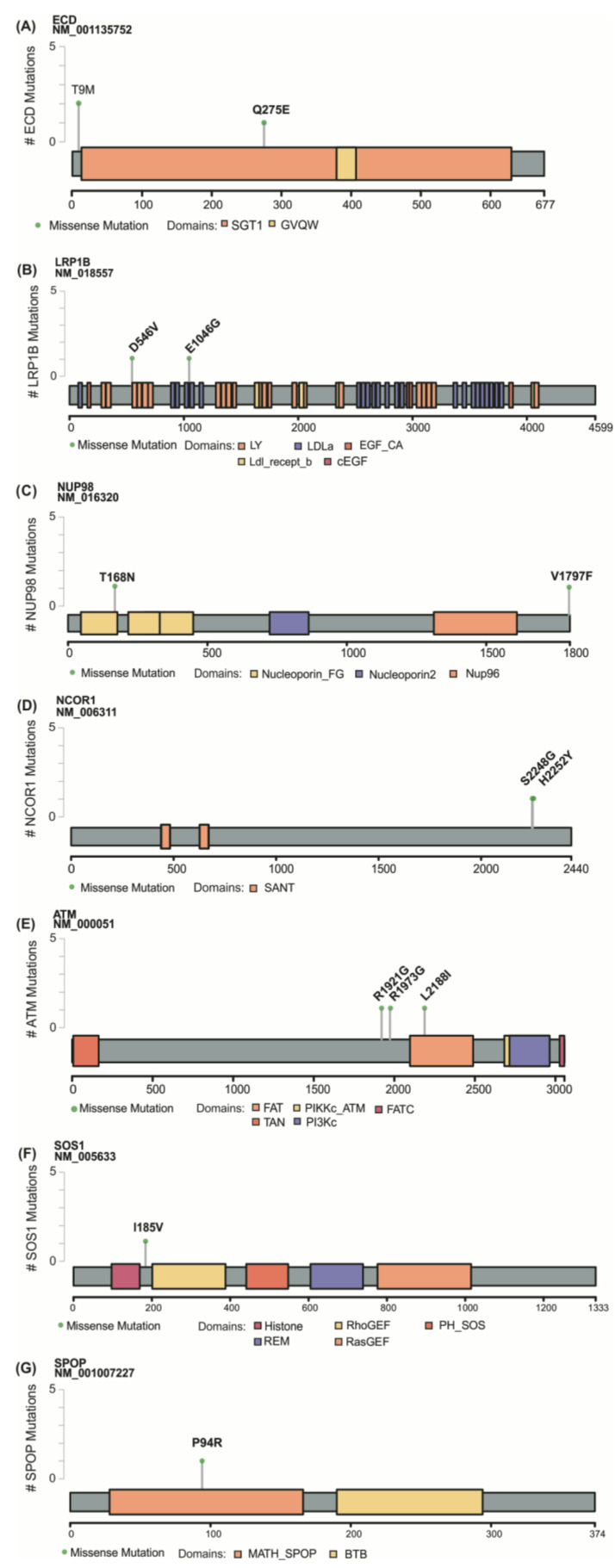
Schematic representation of the proteins encoded by the mutated genes. The amino acid substitutions in ECD (**A**), LRP1B (**B**), NUP98 (**C**), NCOR1 (**D**), ATM (**E**), SOS1 (**F**) and SPOP (**G**) proteins are marked with a pin. Grid on the left represents the number of samples affected by each mutation, and the bottom grid shows the size of the protein.

*ECD* (Ecdysoneless) was recurrently mutated in 3 FVPTC negative samples (cases 5, 3, and 9) (Figure 2 and Figure 3A). Surveying the TCGA-THCA data, *ECD* was found mutated in one PTC sample also negative for *BRAF* and *RAS* mutations. *NUP98* (Nucleoporin 98 and 96 precursor) gene was mutated in FTC and FVPTC samples (cases 27 and 4).

Among the genes previously implicated in thyroid cancer, *LRP1B* (LDL Receptor Related Protein 1B) was found mutated in FTC and FVPTC negative cases (cases 27 and 8) and *NCOR1* (Nuclear Receptor Corepressor 1) in 2 HCC negative cases (cases 28 and 30) (Figure 2 and Figure 3B–D).

We additionally found variants in the *ATM* (A-T mutated) gene in HCC and FVPTC (cases 2, 5, and 29) (Figure 2 and Figure 3E). In one negative sample, *ATM* co-occurred with an *ECD* variant (case 5). *ATM* variants have already been described in 5 PTC from TCGA cohort, 3 of which co-occurring with *BRAF V600E*.

We also observed genetic alterations in potential drivers such as *SOS1* (Son of sevenless homolog 1) (case 30) and *SPOP* (speckle-type POZ protein) (Case 1) (Figure 2 and Figure 3F,G). The SPOP p.P94R substitution was previously described in one PTC sample from TCGA cohort that was negative for *RAS* and *BRAF* mutations.

Although not all substitutions reported here have equal impact, most of our findings are likely damaging by SIFT and Polyphen. Details on the reported variants and their predicted effects are described in Table S2.

We next surveyed for potential drivers previously described in large DNA and RNA-sequencing studies such TCGA on PTC [15] and the molecular landscape of minimally invasive FTC and FTA samples [10]. Among the 32 genes reported by these studies, only 9 (28%) have been found in our cohort (Figure 2—middle panel). However, alterations in these genes frequently co-occurred with genetic changes in other driver genes. For example, variants in *ZFHX3, TG, TSHR, PPM1D, TP53*, and *EZH1* genes co-occurred with canonical mutations in *RAS* and *BRAF* genes. *HIF1A* variants, which have been described co-occurring with BRAF V600E in TCGA samples, here co-occurred with variants in *ECD* and *NCOR1*. Importantly, genes recently described as drivers in thyroid cancers, such as *EIF1AX, IDH1, PTEN, NF1, CHEK2, RB1, MEN1*, *DICER, SK11, MLL*, and *CDH4*, were not mutated in the 14 negative samples.

### 3.3. Gene Fusions in Thyroid Cancer

We identified novel and known fusions events (Figure 2—bottom panel and Table S3). We confirmed the *PAX8-PPARg* fusion in 3 positive samples (cases 26, 6, and 7) and identified the *PAX8-SYN2* fusion co-occurring with the canonical *PAX8-PPARg* fusion in one FVPTC (case 6). The *MET-TFG* fusion, previously described in a PTC from TCGA, was found in an FVPTC co-occurring with the *ATM* mutation (case 2). The *C10orf112-PLXDC2* is a novel fusion found in one FVPTC sample (case 1). *C10orf68-CCDC7* and *KANSL1-ARL17B* are novel fusions identified in 37% and 63% of samples, respectively (Figure 2).

### 3.4. Technical and Experimental Validation by Sanger

The *ECD, NUP98, NCOR1*, and *SPOP* variants were further validated in the discovery cohort by Sanger Sequencing.

As *NCOR1* variants were described in 2/3 HCC cases and as discrimination of HCA and HCC is the main problem for preoperative diagnosis, they were selected to undergo validation in an independent cohort of HCA and HCC. Remarkably, the NCOR1 p.H2252Y variant was recurrent in 3/16 (18%) HCC but was not found in HCA, suggesting it may be relevant to the pathogenesis of HCC. The p.S2248G NCOR1 variant was found only in case 28 from the discovery cohort. Further analysis of *NCOR1* variants in an expanded cohort and functional analysis will help define its role in the pathogenesis of thyroid cancer.

Since there is pathological and experimental evidence of the role of *SPOP* in the tumorigenesis of a variety of human malignancies and the p.P94R variant was already described in one PTC case from TCGA, here we assessed the prevalence of this variant in additional 34 benign and malignant thyroid lesions (12 PTC, 8 FVPTC, 7 FTC, and 7 FTA). The p.P94R variant was absent in all 34 samples tested, suggesting that it is a rare variant.

### 3.5. In Silico Analysis

We performed in silico predictions on variant’s impact using HOPE and Interactome INSIDER softwares. Regarding *NCOR1* variants, HOPE suggests that the wild type residues are crucial for binding with other molecules and the variants may disrupt their interaction with other proteins and likely affect protein function. Additionally, the p.H2252Y substitution is distinct from all conserved variants for this position and likely alters the function of the protein.

SPOP p.P94R amino acid change was found to be larger than the wild type and located on the surface of the protein, near a highly conserved region, which likely affects its interaction with other molecules. Additionally, the change from a neutral amino acid to a positively charged and less hydrophobic residue likely changed the binding properties, disrupting the MATH domain and leading to possible function disruption. Interactome INSIDER revealed that the p.P94R variant is within the interaction site between SPOP and DAXX, PIAS1, GIT2, SRRM1, and ZBTB16 proteins.

### 3.6. Negative Tumors Have Distinctive Expression Profiles

**Figure 4:**
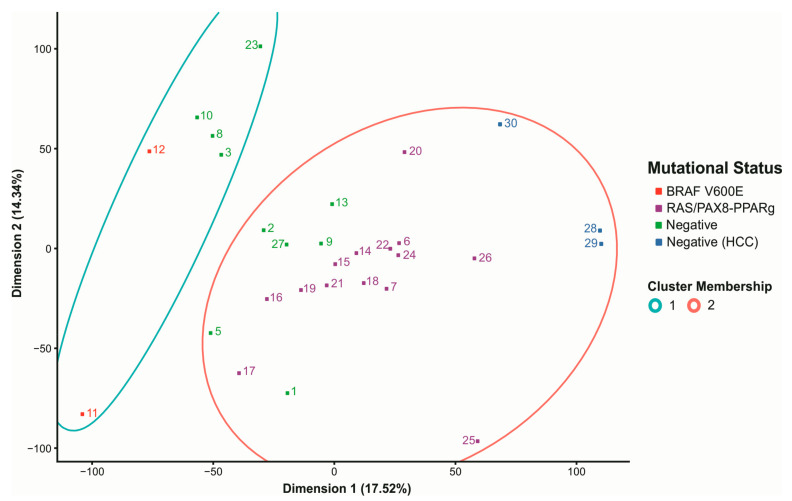
Characterization of the expression profile of the 30-sample dataset. PCA visualization of a hierarchical clustering separates samples according to their expression profile in 2 distinct groups: cluster 1 (blue circle) and cluster 2 (red circle). *BRAF* positive samples (red) are located within cluster 1. *RAS* and *PAX8-PPARg* positive samples (purple) are located within cluster 2. The 14 negative samples (green and dark blue) were split within cluster 1 and 2. Each number represents a sample with a status specific color. X and *Y*-axis represent PCA dimensions.

To characterize the expression profile of the 14 negative samples, we performed a cluster analysis via principal component analysis (PCA), separating all 30 samples according to their expression pattern (Figure 4). The algorithm classified samples in two distinct clusters, distinctively separating *BRAF V600E* positive samples (cluster 1) from *RAS* and *PAX8-PPARg* positive samples (Cluster 2). We not only observed differences in the measured variables between groups but also verified that the negative samples were classified within these two main clusters. Hence, the expression profile of four negative samples visible within cluster 1 resembles that observed for *BRAF V600E*-Like tumors (hereafter called negative BL), whereas the expression profile of six negative samples visible within cluster 2 resembles that observed for *RAS*-Like tumors (hereafter called negative RL). Three negative samples grouped within the opposed margin of cluster 2 are HCC and were considered a distinct group and analyzed independently. Only one negative sample that clustered out was considered an outlier and excluded from this analysis (Figure 4).

**Figure 5:**
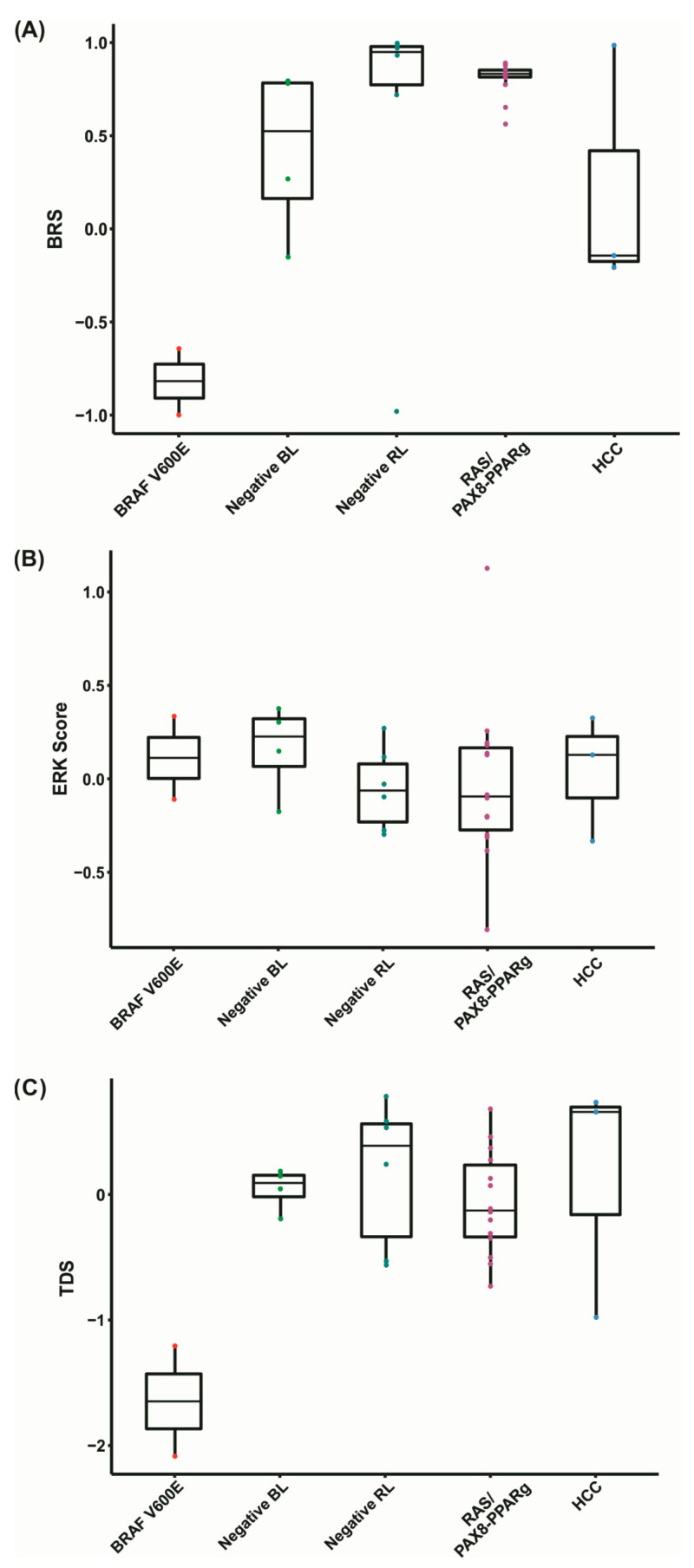
Analysis of BRS, ERK, and TDS scores. (**A**) BRAF V600E-RAS Score (BRS) graphic representation. The closer to −1, the more the expression pattern resembles that of a *BRAF V600E* positive sample and the closer to 1, the more the expression pattern resembles that of a *RAS* positive sample; (**B**) ERK score, based on 52 genes involved in the activation of the MAPK pathway. The higher the score, the more genes activated within this pathway. (**C**) Thyroid Differentiation Score (TDS), based on the expression of 16 thyroid-related metabolism genes. The lower the TDS score, the more dedifferentiated the samples are.

The samples were categorized in 5 groups according to cluster classification and mutational status. Negative samples were classified according to their placement on the hierarchical clustering analysis, i.e., negative RL or negative BL (Figure 4, Table 1). Next, we applied the BRAF V600E-RAS score (BRS), ERK score and thyroid differentiation score (TDS) (Figure 5).

The BRS, which assesses the similarity of a sample’s expression pattern to that of a *BRAF V600E* or *RAS* mutant, is conventionally associated with mutational status, TDS score, and tumor histology. In fact, we found that the BRS discriminated well between *BRAF V600E* and *RAS/PAX8-PPARg* positive samples. The negative RL showed a pattern similar to *RAS/PAX8-PPARg* positive samples. These data suggest that these tumors have a genetic profiling that resembles that driven by *RAS* mutation. However, the negative BL and negative HCC showed a less homogenous pattern. As BRS is a continuous scale ranging from −1 to 1, they were considered weakly correlated with *BRAF V600E*, which is also consistent with the mutations found in follicular-patterned lesions (Figure 5A).

The ERK score, which measures the activation of the ERK-related MAPK pathway, was higher in *BRAF V600E*, negative BL samples, and negative HCC. These data suggests that the mutations found in these tumors might not respond to ERK feedbacks, resulting in high MAPK signaling. On the other hand, the negative RL samples and *RAS/PAX8-PPARg* showed lower ERK scores, suggesting that the mutations found in the negative RL samples may respond to ERK feedback (Figure 5B).

Finally, we measured the TDS, which is based on the expression of 16 thyroid-related genes. As expected, the TDS score was strongly associated with BRS. It was lower in *BRAF V600E* positive samples, compared to the *RAS/PAX8-PPARg* positive samples. Most negative samples showed a TDS comparable to the *RAS/PAX8-PPARg* positive group (Figure 5C).

### 3.7. Negative BL Tumors Have Lower TMB Compared to Negative RL

**Figure 6:**
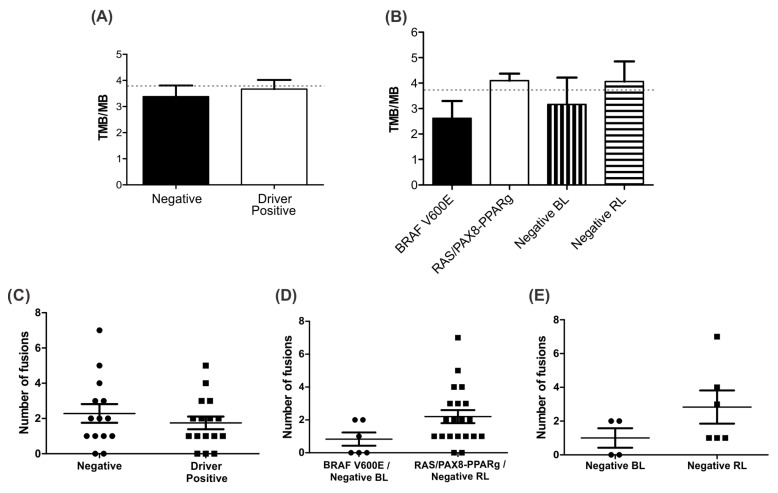
Tumor Mutation Burden Analysis. (**A**) Bar plot representing the TMB/MB calculated for driver negative and driver positive (*BRAF*, *RAS*, and *PAX8-PPARg*) samples. The dotted line represents the general median calculated for all 30 samples; (**B**) bar plot representing the TMB/MB mean per group. The dotted line represents the general median calculated for all 30 samples; (**C**) dot plot representing the number of fusion events in driver positive samples (*BRAF*, *RAS*, and *PAX8-PPARg*) and driver negative samples; (**D**) dot plot representing the number of fusion events in a pool of *BRAF* positive and negative BL samples (*BRAF V600E*/Negative BL) and a pool of *RAS* or *PAX8-PPARg* positive and negative RL samples (*RAS/PAX8-PPARg*/Negative RL); (**E**) dot plot representing the number of fusion events in negative BL and negative RL samples.

The mean TMB for all 30 samples was 3.79. Negative BL samples had a mean TMB of 2.94 while *BRAF V600E* positive samples had a mean TBM of 2.62, which are lower than the general group media. The mean TMB for negative RL samples was 4.59 whereas for the *RAS/PAX8-PPARg* positive it was 4.26, higher than the general group media (Figure 6A,B).

In addition, the 14 negative samples presented a higher range of fusion events when compared to the *BRAF V600E* and *RAS/PAX8-PPARg* positive samples. However, within the negative group, a higher number of fusions are present on negative RL samples. Negative BL samples have a mean of 1.0 fusion/sample compared to a mean of 2.8 fusions/sample in negative RL samples (Figure 6C–E).

### 3.8. Identification of Differentially Expressed Genes

To assess the differences in the expression patterns of the negative samples, we performed a differential expression analysis between negative BL and negative RL groups. We identified a total of 2655 differentially expressed genes (DEGs), 1685 being up-regulated and 970 down-regulated.

Next, to narrow down the pathways that characterize the negative samples, we also performed a differential expression analysis between negative RL samples and *RAS/PAX8-PPARg* positive samples. For that, we analyzed the 6 negative RL samples visible within cluster 2, excluding HCC (Figure 5) versus 14 *RAS/PAX8-PPARg* positive samples. A total of 793 genes were found differentially expressed, 410 being up-regulated and 383 down-regulated. Unfortunately, due to the low number of BRAF positive samples, we were not able to perform a similar analysis on negative BL samples.

### 3.9. Negative BL Tumors Have a High Expression of Immune System Components

In order to gain insight into the biological functions of the 2655 DEGs among negative thyroid carcinomas samples, we performed a gene set enrichment analysis (KEGG and Reactome). Up-regulated genes in negative BL samples are mostly enriched for immune system pathways, compared to negative RL samples (Figure S1A,B). Based on CIBERSORTx, an in silico strategy that employs a machine learning model to estimate the abundance of cell types in a mixed cell population by gene expression data, we were able to infer the fraction of 22 different immune cells in each of our samples.

**Figure 7:**
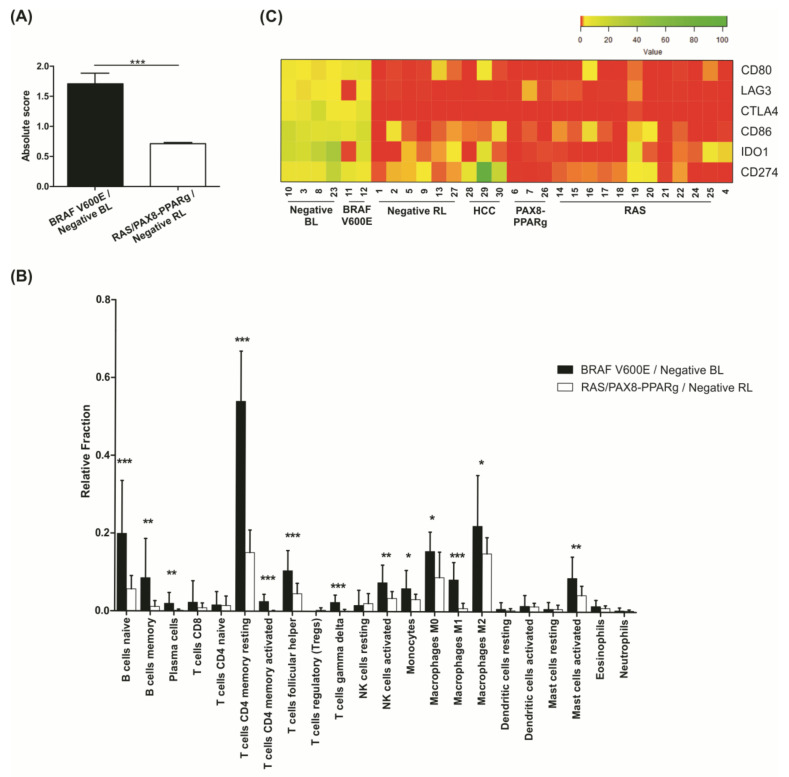
In silico analysis of tumor infiltrating immune cells. (**A**) Absolute score generated by CIBERSORTx software representing the abundance of immune cells in each sample group. The higher the score, the more relative presence of immune cells in that sample. The black bar represent *BRAF* positive and negative BL samples pooled together and the white bar represent *RAS* and *PAX8-PPARg* positive and negative RL samples pooled together; (**B**) fractions of 22 different immune cell subtypes in each sample obtained by CIBERSORTx software. The black bars represent *BRAF* positive and negative BL samples pooled together and the white bars represent *RAS* and *PAX8-PPARg* positive and negative RL samples pooled together; (**C**) heatmap representation of normalized FPM values for immune evasion genes in all samples. * *p* < 0.05; ** *p* < 0.01; *** *p* < 0.001.

As demonstrated by the pathway enrichment analysis, negative BL samples showed a higher fraction of Tumor Infiltrating Immune Cells (TIICs; absolute score mean of 1.82), similar to what was obtained for *BRAF V600E* positive samples (absolute score mean of 1.69). On the other hand, the negative RL samples had a lower fraction of immune cells (absolute score mean 0.71), more similar to the *RAS/PAX8-PPARg* positive samples (absolute score mean 0.70) (Figure 7A and Figure S2A). The immune cell subtype with the higher fraction in *BRAF V600E* positive and negative BL groups is the T cell CD4 memory resting subtype (Figure 7B).

The analysis of 353 PTC samples from TCGA cohort under the same parameters on CIBERSORTx also found a high fraction of TIICs in all samples. Similar to our findings, *BRAF* mutated samples also have a higher absolute score when compared to *RAS* mutated samples (Figure S2B,C).

Regarding gene expression, cancer immune evasion-related genes were found up-regulated in the negative BL samples, such as *CD274* (alias *PD-L1*), *IDO1, LAG-3, CTLA4*, and *CD80* and *CD86* genes, required for the activation of the inhibitory activity of CTLA4. Notably, this expression pattern was similar for *BRAF V600E* positive samples (Figure 7C). Interestingly, negative HCC showed higher levels of *CD274*.

### 3.10. Cancer-Related Pathways Are Differently Regulated in Negative BL and RL Tumors

It is well established that PTC is MAPK-driven with distinct consequences depending whether the tumor harbors *BRAF V600E* or *RAS* mutations. Pathway analysis was used to find specific signaling pathways associated with the genes differentially expressed between analyzed groups. We found that some genes are highly up-regulated, suggesting their related pathways might be activated.

**Figure 8:**
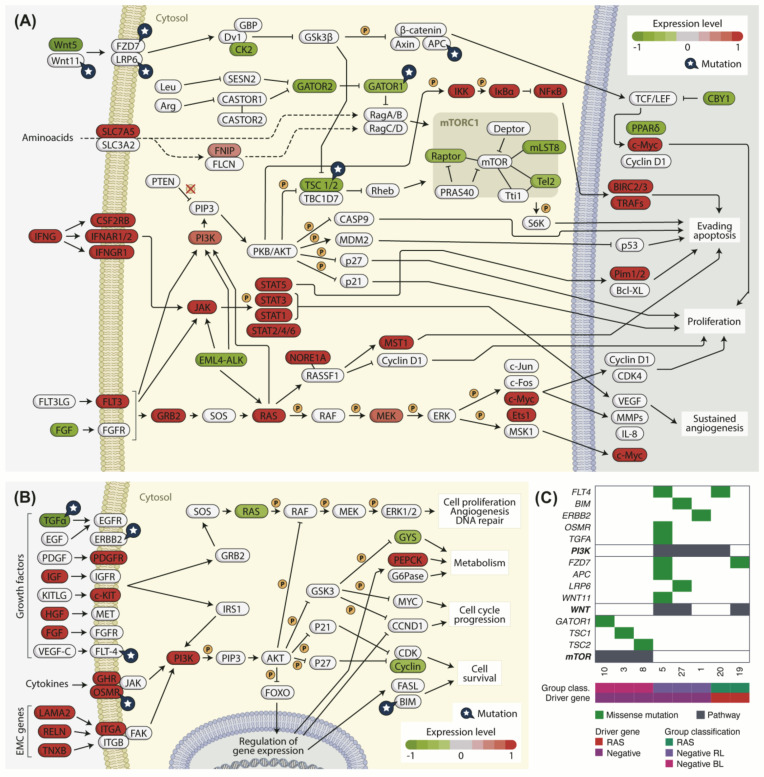
Signaling pathways on negative samples. Genes are marked in shades of red when up-regulated and in shades of green when down-regulated. Mutated genes are marked with a blue star. (**A**) Representation map of tumor-related signaling pathways differentially expressed between negative BL and negative RL samples; (**B**) representation map of PI3K and ERK signaling pathways evidencing DEGs between negative RL and *RAS/PAX8-PPARg* positive samples; (**C**) panel of mutated genes from PI3K, WNT, and mTOR pathways in negative samples. The pathway lines (black) mark which samples are mutated for each pathway.

Similar to that observed for TCGA’s *BRAF V600E* positive samples (Figure S3A,B), negative BL samples had an overall higher expression of cancer-related genes and pathways when compared to negative RL, particularly the PI3K, JAK/STAT, and MAPK pathways (Figure 8A).

Curiously, pathway enrichment on DEGs between negative RL and *RAS/PAX8-PPARg* positive samples evidenced an increase in expression of PI3K pathway related genes in negative RL samples, including important membrane receptors such as *PDGFR*, *c-KIT*, *GHR*, *OSMR*, and *ITGA* (Figure 8B). Alterations in the PI3K pathway may be involved in tumor development on the absence of *RAS* mutations in these samples. The MAPK pathway, however, is similarly expressed in both negative RL and *RAS/PAX8-PPARg* groups (Figure S3C).

As the enrichment analysis revealed that negative samples showed DEG associated with cancer specific pathways, using variant calling data, we further searched for variants (SNVs and InDels) within genes associated with the enriched pathways. We observed alterations in candidate drivers previously described in other cohorts related to PI3K (*FLT4, BIM, ERBB2*, *OSMR*, and *TGFA*), WNT (*FZD7*, *APC*, *LRP6* and *WNT11*), and mTOR (*DEPDC5, TSC1*, and *TSC2*) pathways (Figure 8A–C and Table S2). Mutations in *APC, WNT11, DEPDC5, TSC1, TSC2, ERBB2, OSMR*, and *FLT4* overlapped with RAS or *BRAF* mutations in TCGA-THCA cohort. Whether these additional mutations alter the biological behavior of the tumors still needs further investigation.

## 4. Discussion

Fine needle aspiration (FNA) cytology is an important diagnostic tool for the evaluation of thyroid nodules. The preoperative diagnosis is accurate for about 70% of the cases, while nearly 30% of thyroid nodules fall into the indeterminate category. While molecular tests improved the preoperative diagnosis of thyroid nodules and helped predict prognosis, the clinical management of nodules with indeterminate diagnosis and negative for genetic alterations on known driver genes is still a challenge.

In this study, we characterized the mutational status and expression pattern of thyroid carcinomas that are a source of diagnostic error on FNA and that are negative for the genetic alterations in the top ranked thyroid cancer driver genes. Although the identification of drivers that are infrequently mutated is more difficult, we identified novel candidate driver genes in thyroid cancer. It is important to mention that driver negative samples comprise a small, yet relevant subset of thyroid tumors that include samples that are an important source of diagnostic error on FNA such as FVPTC, FTC, and HCC.

The *ECD* gene was found mutated in three negative samples. It is a cell cycle regulator, involved in cell cycle progression through facilitation of Rb-E2F dissociation and stabilization of p53 through ubiquitination control [40]. In fact, *Ecd* siRNA expression led to a reduction in the level of hyper-phosphorylated Rb, and most genes that were down-regulated upon *Ecd* knockdown were E2F target genes [41,42]. Additionally, overexpression of *ECD* was described in different types of tumors such as breast and pancreatic cancer and correlated with poor prognosis and shorter survival of patients [41,43,44]. Functional analysis demonstrated that in cooperation with driver genes such as *HRAS*, it promoted migration, invasion, and anchorage-independent growth in mammalian epithelial cells and promoted tumor formation in vivo [41]. Others have showed that *ECD* knockdown reduced cell growth, proliferation and tumorigenicity in vivo while affecting glucose uptake by pancreatic cancer cells and reduced phosphorylation of Akt [43]. Although the role of *ECD* variants in thyroid cancer still needs further investigation, 118 variants have been described in the integrative Onco Genomics analysis (intOgen https://www.intogen.org/, accessed in 14 March 2021) software.

We also report *NUP98* and *LRP1B* variants in FTC and FVPTC. *NUP98* variants have not been previously reported in thyroid cancer. Although little is known about NUP98 mutation in thyroid cancer, it is known that it mediates selective transport of RNA molecules between the cytoplasm and nucleus and also regulates cell cycle progression through interactions with several cofactors. Over 30 *NUP98* fusion oncoproteins were described in a spectrum of hematologic malignancies with poor prognosis [45,46].

Regarding *LRP1B,* different variants have already been described in PTC and ATC [47]. Others found somatic mutations and genomic loss of *LRP1B* in thyroid cancer cell lines and thyroid carcinomas correlated with vascular invasion. *LRP1B* ectopic expression inhibited thyroid cancer cell growth and invasion [48]. Additionally, *LRP1B* have been described as a bona fide driver gene in many tumors, including liver, melanoma, colon, lung, gastric, ovarian, and breast [49]. It was one of the most recurrently mutated melanoma genes and was associated with chemoresistance and poor outcome in glioblastoma [50,51]. Importantly, in association with *LRP1B* mutation, higher TMB and improved outcomes in patients treated by immune checkpoint inhibitors across different cancer types have been described [52,53]. Also, concomitant mutations of *KRAS* and *LRP1B* were associated with worse disease-free survival in pancreatic ductal adenocarcinoma [54]. However, the mechanism through which *LRP1B* induces high TMB in tumors is still unknown.

Remarkably, we here found *NCOR1* recurrently mutated in HCC. The p.H2252Y variant was further validated in additional 3 HCC cases while it was not found in HCA. HOPE analysis described this variant as possibly leading to failure in interaction with C1D, an apoptosis-inducing protein.

It is known that *NCOR1* has unique roles in the regulation of thyroid hormone signaling in specific cell types [55]. It has been suggested that *NCOR1*, via protein–protein interaction, is a novel regulator of PI3K signaling and could modulate thyroid tumor progression [56]. *NCOR1* was also found mutated, lost, or with aberrant localization in several human cancers such as bladder, breast, prostate, retinoblastoma, and malignant melanoma. It has also been found deleted in different tumors by TCGA [57] and mutated in PTC, FTC, and ATC by other groups [47]. Functional analysis demonstrated that *NCOR1* is required for colorectal cancer cell growth and protects against cellular senescence, while loss of *NCOR1* reduced tumorigenic growth in vivo [58]. In addition to its role in cell cycle regulation, *NCOR1* was identified as a central transcriptional repressor check point of genes involved in inflammation [58]. *NCOR1* was also involved in regulation of mitochondrial function and ROS generation [52] and required to maintain systemic metabolic homeostasis in vivo [59]. Its alteration has also been associated with enhanced tumor immunogenicity and higher TMB in patients with bladder cancer.

Altogether, our data suggest that *NCOR1* may play role in the pathogenesis of HCC. Whether *NCOR1* also play a role in the pathogenesis of other thyroid cancer subtypes needs further investigation.

The recurrently mutated gene *ATM* is a serine/threonine kinase that plays a role in DNA repair of double strand breaks. Although the variants reported here have not been previously described, they are located near the site of activation of the protein and were classified as damaging by Polyphen and Sift scores [60]. *ATM* is another gene potentially associated with higher TMB and increased expression levels of some immune checkpoints [61]. Remarkably, most negative samples classified as RL, with higher TMB, presented *LRP1B, NCOR1*, and *ATM* variants.

*SOS1* and *SPOP* have been previously found mutated in thyroid tumors negative for *RAS* and *BRAF* mutations and in several types of cancers such as prostate, lung, and colon.

It has been shown that *SPOP* suppress tumorigenesis in human cancers via regulation of cell growth, apoptosis, migration, invasion, and drug resistance by targeting different downstream substrates [62]. As *SPOP* may play a dual role in cancer, it has been suggested that it exerts its biological function in a cancer type-specific manner [62]. Despite *SPOP* being the most commonly mutated tumor suppressor gene in prostate and its variants being highly prevalent in other human cancers, the p.P94R variant has been recurrently found only in thyroid tumors and its role has yet to be determined [62]. In silico analysis has shown that the mutant residue is in the surface of the protein, in a highly conserved region, and the drastic changes in amino acid properties may lead to failure in interaction. Furthermore, the P94 position is within the interaction site of SPOP with several proteins, including DAXX (death domain-associated protein), which may be disrupted by the p.P94R variant. Interestingly, DAXX overexpression in cancer is related to disease progression and treatment resistance. It has been shown that in kidney cancer, SPOP promoted the ubiquitination and degradation of DAXX [62]. Failure of interaction between them will compromise DAXX degradation and lead to its accumulation in the cell [63,64]. Interestingly, DAXX has also been associated with degradation of Gli2 and PTEN in kidney cancer [62].

Although this variant has been described in benign lesions, it was described in PTC and minimally invasive FTC [10,15,65,66]. To better understand the role of *SPOP*, the p.P94R variant was further validated in an independent set of thyroid benign and malignant thyroid tumors. Although we did not find the SPOP p.P94R variant in an extended validation cohort, the low prevalence of this variant is still in accordance with literature. Altogether, SPOP p.P94R may be an early stage tumor variant that requires cooperation with other drivers in the pathogenesis of thyroid tumors and so we believe it should be further investigated.

We have previously screened out the most common fusions found in thyroid cancer and identified four novel fusions that have drawn our attention. While *SYN2* has already been described fused with *PPARg* [67], here we identified *PAX8* as a new partner. *C10orf112-PLXDC2* fusion, recently associated with the progression of meningioma [68], co-occurred in one sample with *SPOP* variant. *C10orf68-CCDC7* was identified co-occurring with other variants across most samples. Interestingly this fusion showed relatively high recurrence in invasive breast carcinoma, prostate, kidney, and lung [69]. *KANSL1-ARL17B* has been considered a polymorphic translocation with a possible role as a predisposition marker [70]. Interestingly, multiple read-through fusion transcripts or even fusions that occur through trans-splicing mechanism have been described in several normal cells [71]. Although the role of these fusions still needs to be determined, they are highly recurrent and may have a role in cell function.

Despite the fact that our study brings novel candidate driver genes that might be associated with pathogenesis or progression of thyroid tumors, we understand that it has some limitations such as sample size. Additionally, although some variants work as drivers in certain tumor subtypes while being permissive in other subtypes, functional analysis will certainly help fill these gaps. However, as some of these genes co-occur with other driver genes in different tumor subtypes, mutational screening of candidate drivers in a large set of thyroid tumors is required to select which ones must undergo functional analysis and in which genetic background. Functional analysis performed in these candidate genes without further analysis could lead to a biased effect in cell lines and likely conflicting results.

Regarding the expression profiling, hierarchical clustering on principal components separated the negative samples into two distinct groups, named RL and BL, each of which contained samples with *RAS* or *BRAF V600E* mutations.

The expression signature presented in this work was comparable to that observed in the TCGA cohort and available database, reinforcing our results despite the overall low number of samples in our cohort. Moreover, although there are only two *BRAF V600E* positive samples in our cohort, we believe them to be true representatives of their group as they behaved similarly to TCGA *BRAF V600E* samples across all analysis performed in this study. Our set of samples showed BRS and TDS, immune infiltration rate, and level of MAPK pathway expression similar to that observed in TCGA cohort with *BRAF V600E* mutation.

Negative BL and RL samples behaved similarly to the negative samples of the TCGA-THCA study on BRS and TDS scores [15]. TCGA showed that samples harboring fusions with genes such as *ALK, MET*, and *NTRK1/3* and negative samples had an intermediary or *RAS*-like BRS and a higher TDS. Furthermore, they demonstrated that other mutations in *BRAF* gene scored closer to *RAS* positive samples in both scores. On ERK score analysis, negative BL samples showed higher activation of ERK-related MAPK pathway, compared to negative RL samples and these data are confirmed by differential expression analysis between negative BL and negative RL samples. The negative BL tumors may harbor mutations that are more sensitive to the negative feedback regulation of the ERK1/2 than negative RL tumors. This shows that driver negative samples are not a homogeneous group and may behave differently and benefit from different clinical approaches. Altogether, these findings highlights that the negative samples have distinct expression profiling and, therefore, likely activate/deactivate distinct cancer-related pathways.

When we investigated the pathways that are dysregulated in negative samples, we observed that genes that encoded proteins that control the mTOR pathway are mutated or down-regulated in negative BL samples. The mTOR pathway has been found up-regulated in the presence of *BRAF V600E* mutation, which might cooperate with loss of expression of the *LKB1* gene, leading to failure in activating the TSC1/2 complex and activation of mTOR pathway [72,73]. We have found mutations in genes *TSC1*, *TSC*2, and *DEPDC5* (*GATOR 1*) and down-regulation of *TSC2* and *NPR2* (part of GATOR1 complex) in negative BL samples. These genes may play a role in the activation of the mTOR pathway in the absence of other commonly found alterations. In addition, these findings may partly explain the difference observer in ERK score for the negative BL samples.

The topmost DEGs between negative BL and negative RL samples belonged to immune system pathways. Interestingly, we found that negative BL samples have a higher expression of immune components and *CD274* (*PD-L1*), *IDO1*, and *CTLA-4* immune evasion genes. The same result was observed for *BRAF* positive samples from the TCGA cohort. Several studies already described an abundant presence of immune cells in *BRAF V600E* positive thyroid tumors with more aggressive behavior, as well as higher expression of immune evasion genes such as *PD-L1*, *IDO1*, and *CTLA-4* [74,75]. They suggested that as PTC develops, the proportion of tumor infiltrating immune cells increases significantly, especially in *BRAF V600E* positive cells [75].

Frequently, higher TMB is associated with better response to immune checkpoint inhibitors (ICI), however this is not a universal rule [76]. This happens because ICI treatments only re-invigorate immune cells, but do not induce their formation. Based on that, it has been proposed to predict ICI efficacy based on expression of T-cell markers and Interferon signature, as ICI treatment will only succeed if effector immune cells are presents in the tumor microenvironment [76]. Although the negative BL samples have a low TMB, there is a high presence of immune cells and the immune evasion genes seen overexpressed in our samples are common targets for immunotherapy in many tumors using ICI [77]. We believe this finding is of great interest, as the portrait in the immune landscape observed in *BRAF V600E* and negative BL samples might be therapeutically relevant.

When looking at DEGs between Negative RL and *RAS/PAX8-PPARg* positive samples, the main difference was the pronounced expression of genes in PI3K/AKT pathway. In cancer, the common drivers for this pathway are activating mutations in PIK3CA gene, inactivating modifications in PTEN, and activating modifications in AKT isoforms or in PI3K activating receptor tyrosine kinases (RTKs) [78]. We found mutations in RTK genes *ERBB2* (also known as *HER2*) and *FLT4* (also known as *VEGFR-3*) and cytokine receptor *OSMR* in negative RL samples that have been previously described as able to activate the PI3K/AKT pathway [78,79]. These mutations may explain the activation of this pathway in the absence of *RAS* mutations and highlight the importance of further investigation.

## 5. Conclusions

We here explored the molecular profiling of thyroid carcinomas that are negative for the main thyroid driver genes and the main source of preoperative diagnostic errors. We identified recurrent genetic alterations in candidate drivers in 92% (13/14) of negative samples, which were validated by Sanger. The list includes known drivers that had not been associated with thyroid cancer such as *ECD* and *NUP98* and recovers other potential drivers previously described in thyroid tumors such as *LRP1B, NCOR1, ATM, SOS1*, and *SPOP*. In silico analysis confirmed the potential of most of the predicted variants, particularly *NCOR1* and *SPOP*. Negative samples showed distinct expression profiling; however, they resemble those observed in *BRAF V600E* or *RAS* positive tumors in many different aspects, with highlights to tumor infiltrating immune cells. In summary, our results not only increase knowledge of the molecular pathogenesis of driver negative thyroid tumors but also describe novel variants in thyroid cancer, which may ultimately help to narrow down the dark matter, i.e., the driver negative samples. These data might impact preoperative diagnosis and management of thyroid nodules commonly classified as indeterminate on FNA analysis, provide directions for further experimental validation analysis and might represent suitable candidates for adjuvant therapy.

## Figures and Tables

**Table 1:** Clinical pathological data.

Sample	Tumor Type	Mutational Status	Expression Classifier Group	Gender	Age at Surgery	Tumor Size	Multifocality	ETE	Vascular Invasion	Lymph Node Metastasis	Stage	Risk
1	FVPTC	Negative	Negative RL	F	45	3.0	N	N	N	N	I	Low
2	FVPTC	Negative	Negative RL	F	31	3.2	N	N	Y	Y	I	High
3	FVPTC	Negative	Negative BL	F	76	1.0	Y	N	N	Y	II	High
4	FVPTC	Negative	-	F	68	8.0	Y	Y	Y	N	IVA	High
5	FVPTC	Negative	Negative RL	F	52	0.9	N	N	N	N	I	Low
6	FVPTC	Positive	*RAS/PAX8-PPARg*	M	68	7.5	N	N	N	N	II	High
7	FVPTC	Positive	*RAS/PAX8-PPARg*	F	53	4.0	Y	N	N	N	I	Low
8	FVPTC	Negative	Negative BL	M	40	8.5	Y	N	Y	Y	I	High
9	FVPTC	Negative	Negative RL	F	73	1.2	Y	N	N	N	I	Low
10	CVPTC	Negative	Negative BL	F	59	2.5	Y	N	N	N	I	Low
11	CVPTC	Positive	BRAF V600E	F	46	1.7	N	Y	Y	Y	I	High
12	CVPTC	Positive	BRAF V600E	F	41	1.3	N	N	N	N	I	Low
13	CVPTC	Negative	Negative RL	F	56	3.0	Y	Y	N	Y	III	High
14	FVPTC	Positive	*RAS/PAX8-PPARg*	M	32	5.0	N	Y	N	N	I	High
15	FVPTC	Positive	*RAS/PAX8-PPARg*	M	40	4.0	N	N	N	N	I	Low
16	FVPTC	Positive	*RAS/PAX8-PPARg*	F	28	5.0	Y	N	N	N	I	Low
17	FVPTC	Positive	*RAS/PAX8-PPARg*	F	55	1.7	Y	N	N	N	I	Low
18	FVPTC	Positive	*RAS/PAX8-PPARg*	F	36	3.0	N	N	N	N	I	Low
19	FVPTC	Positive	*RAS/PAX8-PPARg*	F	45	1.1	N	N	N	N	I	Low
20	FTC	Positive	*RAS/PAX8-PPARg*	F	45	3.5	Y	Y	N	N	II	High
21	FTC	Positive	*RAS/PAX8-PPARg*	F	48	3.2	N	N	Y	N	I	High
22	FTC	Positive	*RAS/PAX8-PPARg*	F	48	1.6	N	Y	Y	N	I	High
23	FTC	Negative	Negative BL	F	35	4.5	N	N	N	N	I	Low
24	FTC	Positive	*RAS/PAX8-PPARg*	F	76	7.5	N	Y	Y	Y	IVA	High
25	FTC	Positive	*RAS/PAX8-PPARg*	M	70	10.3	N	Y	Y	Y	IVA	High
26	FTC	Positive	*RAS/PAX8-PPARg*	F	70	10.0	N	N	N	N	II	High
27	FTC	Negative	Negative RL	F	60	2.5	Y	N	Y	N	IVA	High
28	HCC	Negative	Negative (HCC)	M	NA	NA	NA	NA	NA	NA	NA	NA
29	HCC	Negative	Negative (HCC)	F	70	5.0	N	N	Y	Y	IVA	High
30	HCC	Negative	Negative (HCC)	F	63	6.0	N	N	Y	N	IVA	High

Abbreviations: CVPTC, classic variant of papillary thyroid carcinoma; FVPTC, follicular variant of papillary thyroid carcinoma; FTC, follicular thyroid carcinoma; HCC, Hürthle cell carcinoma; NA, not available; Negative RL, negative Ras-Like; Negative BL, negative BRAF-like; ETE, extrathyroidal extension

## Data Availability

TCGA data used in this project can be accessed at https://portal.gdc.cancer.gov (accessed on 10 July 2020), ENCODE normal thyroid can be found at https://www.encodeproject.org (accessed on 20 February 2019).

## Supplementary Materials

The following are available online at https://www.mdpi.com/article/10.3390/cancers13205184/s1, Figure S1: Ridgeplot representing the most significantly enriched pathways by KEGG or GSEA enrichment analysis, Figure S2: Immune cell fraction, Figure S3: Kegg’s ‘Pathways in cancer’ enriched pathway, Table S1: Summary of RNA-Seq alignment data, Table S2: Genomic specifications of described variants, Table S3: Fusions detected in this study on 30 samples by RNAseq.
