# Supplementary material for: Molecular Signature Expands the Landscape of Driver Negative Thyroid Cancers
Source: Cancers (Basel). 2021 Oct 15;13(20):5184. doi: 10.3390/cancers13205184 (PMC8534197; doi:10.3390/cancers13205184)
Supplement: Supplementary file 1 [file cancers-13-05184-s001.zip › cancers-1388243 supplementary-for xml/cancers-1388243 supplementary-for xml.pdf]

Supplementary Materials: Molecular Signature Expands the Landscape of Driver Negative Thyroid Cancers

Larissa Valdemarin Bim, Thaise Nayane Ribeiro Carneiro, Vanessa Candiotti Buzatto, Gabriel Avelar Colozza-Gama, Fernanda C. Koyama, Debora Mota Dias Thomaz, Ana Carolina de Jesus Paniza, Eunjung Alice Lee, Pedro Alexandre Favoretto Galante and Janete Maria Cerutti

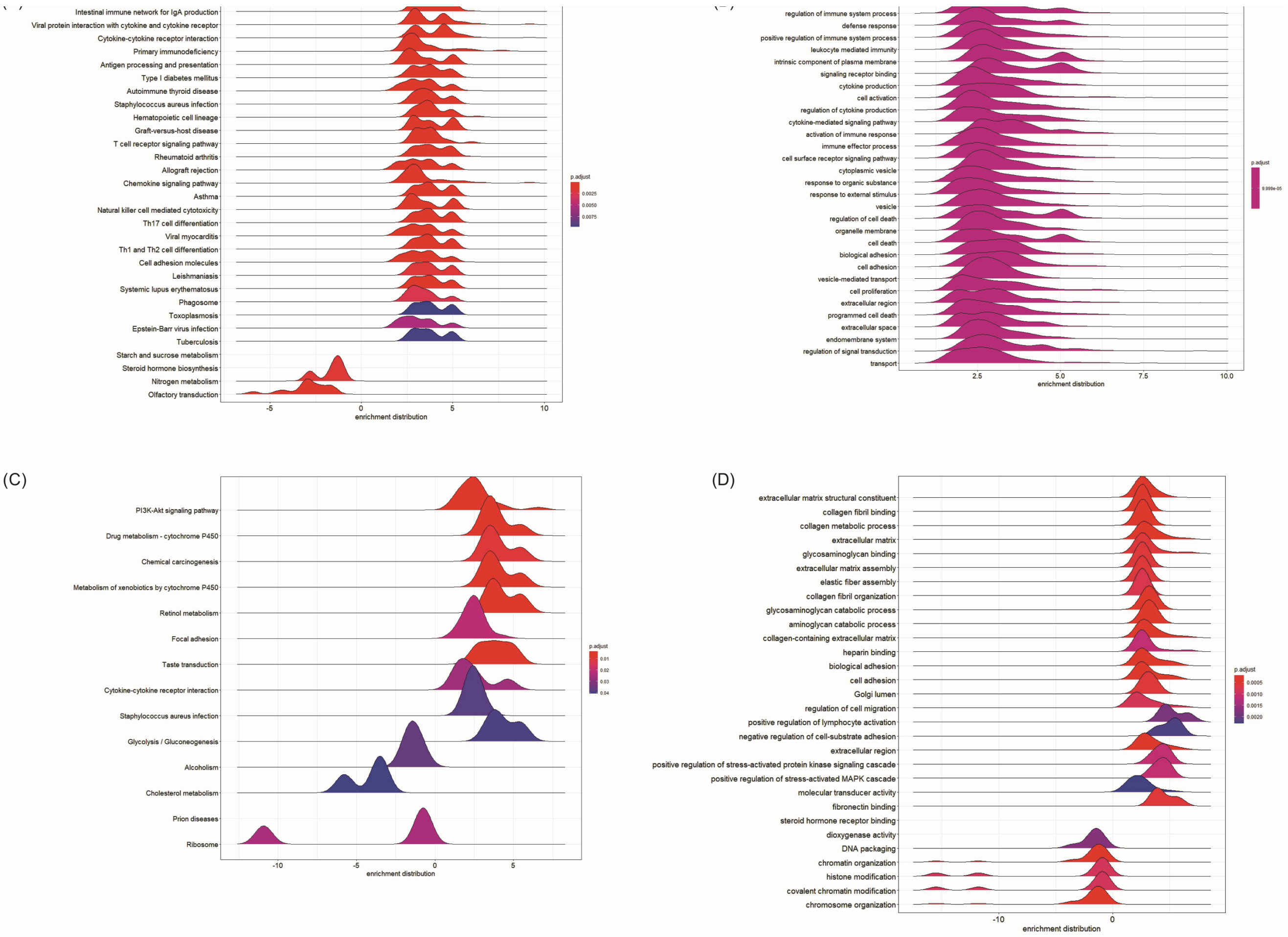

Figure S1. Ridgeplot representing the most significantly enriched pathways by KEGG or GSEA enrichment analysis. A. KEGG and B. GSEA pathway enrichment for DEGs between Negative BL and Negative RL groups; C. KEGG and D. GSEA pathway enrichment for DEGs between Negative RL and RAS/PAX8-PPARg positive groups.

(A)

Input Sample	B cells naive	B cells memory	Plasma cells	T cells CD8	T cells CD4 naive	T cells CD4 memory resting	T cells CD4 memory activated	T cells follicular helper	T cells regulatory (Tregs)	T cells gamma delta	NK cells resting	NK cells activated	Monocytes	Macrophages M0	Macrophages M1	Macrophages M2	Dendritic cells resting	Dendritic cells activated	Mast cells resting	Mast cells activated	Eosinophils	Neutrophils	P-value	Correlation	RMSE	Absolute score (sig.score)
BL_10	0.004	0.264	0.067	0	0	0.632	0	0.109	0	0.049	0	0.108	0.016	0.171	0.129	0.484	0	0	0	0.066	0	0.02	0	0.670	0.782	2.119
BL_3	0.22	0.135	0	0	0.085	0.531	0.052	0.111	0	0.029	0	0.043	0.014	0.107	0.08	0.197	0	0	0	0.072	0	0	0	0.756	0.721	1.676
BL_8	0.329	0.072	0	0	0.01	0.628	0.007	0.172	0	0.011	0	0.12	0.036	0.149	0.077	0.187	0	0.006	0	0.103	0.035	0	0	0.751	0.723	1.943
BL_23	0.253	0	0.011	0	0	0.349	0.032	0.086	0	0.012	0	0.077	0.085	0.214	0.11	0.158	0	0.012	0	0.116	0.023	0.003	0	0.534	0.852	1.541
B_11	0.063	0.002	0	0	0	0.427	0.031	0.016	0	0	0.095	0	0.134	0.089	0	0.159	0.042	0.071	0.044	0	0.002	0	0.002	0.312	0.954	1.176
B_12	0.325	0.039	0.04	0.135	0	0.674	0.029	0.131	0	0.04	0	0.098	0.071	0.2	0.095	0.136	0	0	0	0.164	0.029	0	0	0.776	0.709	2.205
RL_1	0.068	0	0.004	0	0.007	0.138	0	0.067	0	0	0.031	0.034	0.028	0.016	0.004	0.125	0.006	0.022	0.028	0.003	0.015	0.003	0.003	0.256	0.978	0.600
RL_2	0.099	0	0	0	0	0.146	0	0.05	0.013	0.02	0	0.041	0.035	0.137	0.013	0.193	0	0	0	0.058	0.018	0.004	0	0.531	0.853	0.826
RL_5	0.052	0	0.001	0	0.045	0.307	0	0.024	0	0	0.007	0.042	0.029	0.031	0.014	0.148	0	0.003	0	0.075	0.004	0	0	0.570	0.829	0.782
RL_9	0.153	0	0.013	0	0	0.171	0	0.042	0	0	0.055	0.008	0.035	0.033	0.007	0.08	0	0.026	0	0.046	0.009	0.001	0.002	0.289	0.966	0.678
RL_13	0.025	0.017	0	0.014	0.018	0.166	0	0.021	0	0	0.002	0.049	0.034	0.056	0.003	0.164	0.002	0.022	0.024	0.008	0.007	0	0	0.436	0.899	0.631
RL_27	0.043	0	0.003	0.011	0	0.238	0	0.043	0	0	0.004	0.053	0.04	0.061	0.006	0.166	0.003	0.021	0	0.042	0.022	0.008	0	0.502	0.867	0.763
P8_6	0.114	0	0.005	0	0	0.127	0	0.028	0.004	0	0.065	0.019	0.03	0.051	0.001	0.144	0	0.009	0	0.027	0.008	0.005	0.001	0.358	0.935	0.636
P8_7	0.046	0.015	0	0.019	0	0.219	0	0.035	0	0	0	0.044	0.037	0.025	0.008	0.143	0	0.014	0	0.066	0.018	0.009	0.001	0.335	0.946	0.697
P8_26	0.02	0.028	0	0	0	0.081	0	0.045	0.007	0	0.011	0.042	0.011	0.122	0.001	0.107	0.006	0.023	0	0.06	0.015	0.004	0	0.464	0.886	0.581
R_14	0.019	0.011	0.002	0	0.084	0.156	0	0.014	0	0	0.01	0.034	0.023	0.18	0.005	0.136	0	0.005	0	0.047	0	0	0	0.556	0.836	0.725
R_15	0.022	0.013	0.001	0	0.033	0.139	0	0.01	0	0	0.023	0.02	0.013	0.196	0.008	0.199	0.002	0.002	0	0.046	0.006	0	0	0.618	0.798	0.732
R_16	0.07	0	0.004	0.007	0	0.066	0	0.065	0.008	0	0.012	0.03	0.025	0.235	0	0.245	0.011	0.019	0	0.042	0.004	0.003	0	0.592	0.813	0.844
R_17	0.06	0.052	0	0.005	0	0.148	0	0.075	0	0	0	0.054	0.045	0.072	0.003	0.168	0	0.013	0.032	0.038	0	0.002	0	0.437	0.899	0.767
R_18	0.058	0.042	0	0.029	0	0.115	0	0.055	0	0	0	0.06	0.015	0.082	0.009	0.198	0	0.01	0	0.084	0.015	0.008	0.01	0.209	1.002	0.780
R_19	0.08	0	0	0.049	0	0.208	0.001	0.125	0	0.003	0	0.055	0.028	0.074	0.065	0.131	0.001	0.006	0	0.05	0.005	0	0	0.501	0.867	0.881
R_20	0.048	0	0	0.013	0	0.111	0.008	0.078	0	0.001	0	0.042	0.053	0	0.001	0.18	0.025	0.035	0	0.084	0.02	0.009	0	0.367	0.930	0.708
R_21	0.045	0.019	0.002	0	0.009	0.102	0	0.044	0.023	0	0.075	0.004	0.07	0.046	0.005	0.161	0.005	0.01	0.018	0.013	0.016	0	0.01	0.207	0.996	0.668
R_22	0.021	0.011	0.001	0	0.067	0.162	0	0.034	0	0	0.006	0.036	0.019	0.177	0.005	0.096	0	0.017	0	0.043	0	0	0	0.559	0.834	0.695
R_24	0.04	0.004	0.004	0.011	0.017	0.134	0	0.026	0.001	0	0.047	0.016	0.027	0.063	0.008	0.088	0.002	0.008	0.019	0.015	0.014	0.002	0.003	0.238	0.986	0.544
R_25	0.05	0.019	0	0.013	0.009	0.08	0	0.031	0.011	0	0.066	0	0.031	0.1	0.005	0.118	0	0.009	0.017	0.013	0.005	0.004	0.003	0.238	0.986	0.582
4	0.043	0.012	0	0	0.01	0.105	0.005	0.025	0	0	0.035	0.009	0.021	0.124	0.005	0.135	0.006	0.018	0	0.044	0.011	0.015	0.002	0.270	0.976	0.621
H_28	0.022	0.025	0	0.003	0	0.108	0	0.03	0.016	0	0.002	0.055	0.059	0.029	0.012	0.127	0	0.009	0	0.076	0.02	0.004	0	0.398	0.917	0.594
H_29	0.029	0.02	0	0.007	0	0.073	0	0.037	0	0	0	0.044	0.068	0.031	0.001	0.112	0	0.021	0	0.078	0.035	0.008	0	0.424	0.905	0.565
H_30	0.045	0	0	0.002	0	0.173	0	0.048	0	0	0	0.072	0.032	0.072	0.008	0.212	0	0.013	0	0.097	0.013	0.003	0	0.641	0.792	0.790

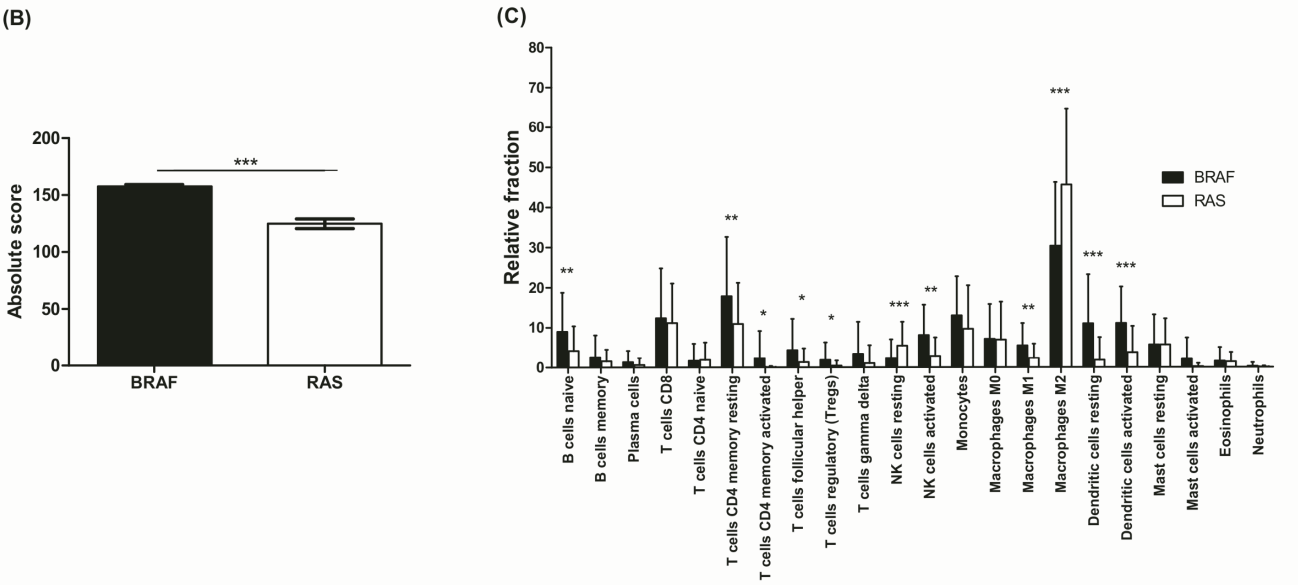

Figure S2. A. Heatmap representation of the fractions of each immune cell subtype in each sample obtained by CIBERSORTx software. The lower fractions have shades of blue and the higher have shades of red; **B.** Fractions of each immune cell subtype in TCGA-THCA's BRAF or RAS positive sample obtained by CIBERSORTx software. Black bars represent *BRAF* mutated samples and white bars represent *RAS* mutated samples; **C.** Absolute score generated by CIBERSORTx software representing the abundance of immune cells in TCGA-THCA's samples. The higher the score, the more presence of immune cells. The black bar represents *BRAF* mutated samples and the white bar represents *RAS* mutated samples.

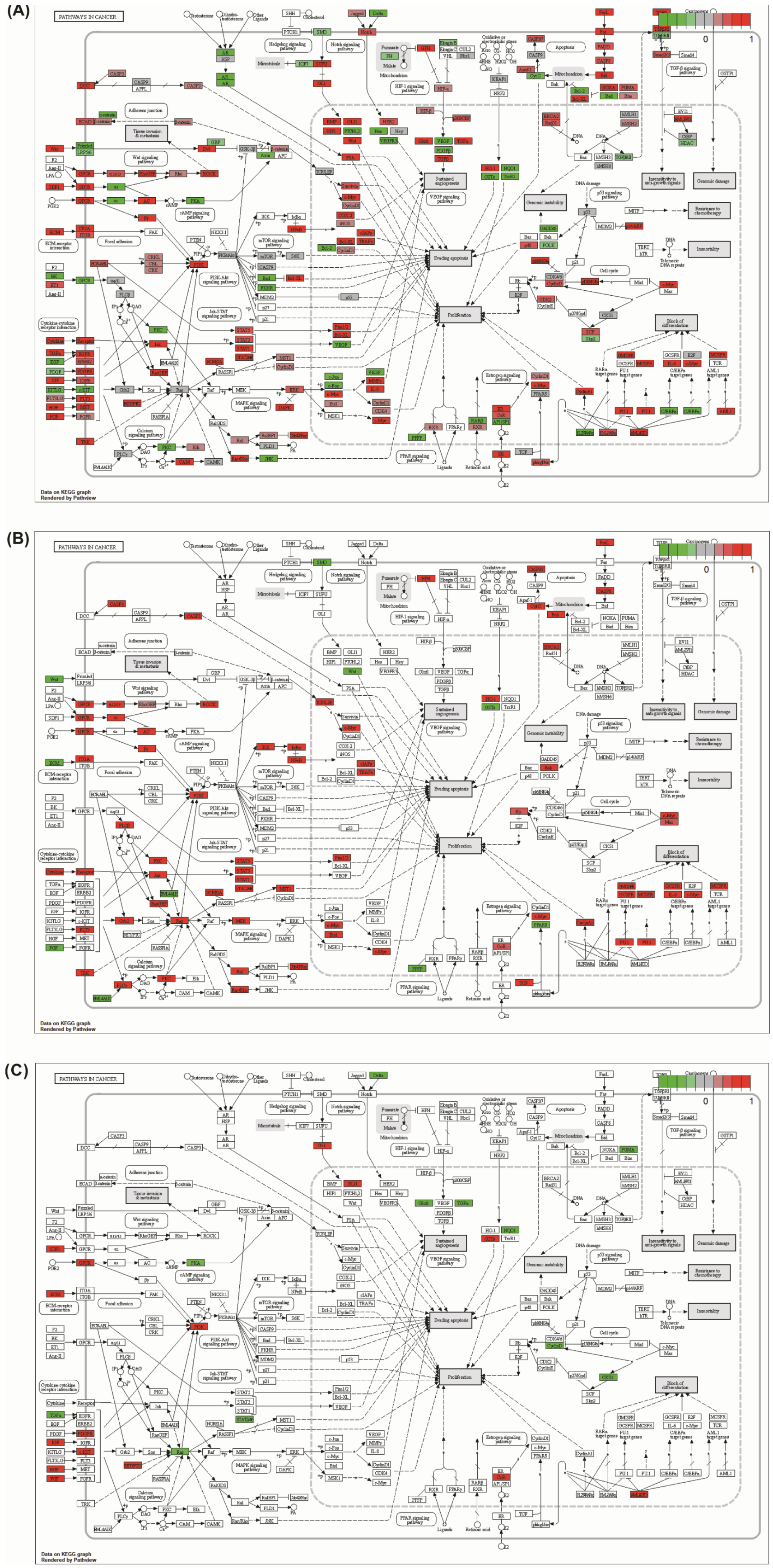
(A)
PATHWAYS IN CANCER
Data on KEGG graph
Rendered by Pathview
(B)
PATHWAYS IN CANCER
Data on KEGG graph
Rendered by Pathview
(C)
PATHWAYS IN CANCER
Data on KEGG graph
Rendered by Pathview

Figure S3. Kegg's 'Pathways in cancer' enriched pathway. Colored squares represent DEG detected in our differential expression analysis. Red boxes represent upregulated genes and green boxes represent downregulated genes, the degree of fold change is expressed in the upper-right legend; **A.** DEGs between TCGA-THCA *BRAF* and *RAS* positive samples; **B.** DEGs between negative BL and negative RL samples; **C.** DEGs between negative RL and RAS/PAX8-PPARg positive samples.
